# A Hierarchical Probabilistic Model for Rapid Object Categorization in Natural Scenes

**DOI:** 10.1371/journal.pone.0020002

**Published:** 2011-05-25

**Authors:** Xiaofu He, Zhiyong Yang, Joe Z. Tsien

**Affiliations:** 1 Brain and Behavior Discovery Institute, Georgia Health Sciences University, Augusta, Georgia, United States of America; 2 Department of Computer Science and Technology, East China Normal University, Shanghai, China; 3 Department of Ophthalmology, Georgia Health Sciences University, Augusta, Georgia, United States of America; 4 Department of Neurology, Georgia Health Sciences University, Augusta, Georgia, United States of America; National Microelectronics Center, Spain

## Abstract

Humans can categorize objects in complex natural scenes within 100–150 ms. This amazing ability of rapid categorization has motivated many computational models. Most of these models require extensive training to obtain a decision boundary in a very high dimensional (e.g., ∼6,000 in a leading model) feature space and often categorize objects in natural scenes by categorizing the context that co-occurs with objects when objects do not occupy large portions of the scenes. It is thus unclear how humans achieve rapid scene categorization.

To address this issue, we developed a hierarchical probabilistic model for rapid object categorization in natural scenes. In this model, a natural object category is represented by a coarse hierarchical probability distribution (PD), which includes PDs of object geometry and spatial configuration of object parts. Object parts are encoded by PDs of a set of natural object structures, each of which is a concatenation of local object features. Rapid categorization is performed as statistical inference. Since the model uses a very small number (∼100) of structures for even complex object categories such as animals and cars, it requires little training and is robust in the presence of large variations within object categories and in their occurrences in natural scenes. Remarkably, we found that the model categorized animals in natural scenes and cars in street scenes with a near human-level performance. We also found that the model located animals and cars in natural scenes, thus overcoming a flaw in many other models which is to categorize objects in natural context by categorizing contextual features. These results suggest that coarse PDs of object categories based on natural object structures and statistical operations on these PDs may underlie the human ability to rapidly categorize scenes.

## Introduction

Humans can remember extraordinarily rich details in thousands of scenes viewed for a very brief period [1]. Humans can also grasp the gist of complex natural scenes very quickly [2], [3], [4]. This is often called rapid scene categorization since it requires little or no attention and top-down feedback plays a limited role. This amazing ability challenges the traditional view of visual information processing in several major ways. In the mainstream framework of vision [5], [6], [7], visual neurons are conceived to perform bottom-up image-based processing (e.g., computing zero-crossings, luminance and texture gradients, stereoscopic and motion correspondence, and grouping) to build a series of symbolic representations (e.g., primal sketch, 2½ dimensional (2½D) sketch, and 3D representation). It is difficult to reconcile this view of visual processing with human performance on rapid scene categorization [8], [9], [10]. On one hand, low-level visual features including edges, junctions, and various image gradients are insufficient for revealing the content of complex natural scenes. On the other hand, the computation needed to build such symbolic representations seems too time-consuming for rapid scene categorization.

This quandary has led to several alternative ideas. One proposal is that the visual system processes global scene features [11], [12], [13], [14], [15] first and then uses the results of global processing to guide local processing [16], [17], [18]. Models that use global features such as the energy of spectra at low spatial frequency perform well on certain tasks of scene categorization [11], [15], but poorly on tasks such as categorizing scenes with animals since these global features are not useful for identifying and localizing objects in natural context.

Another proposal is to formulate scene categorization as a statistical decision process in a high-dimensional space of low-to-intermediate-level visual features without developing a series of symbolic representations [19], [20], [21], [22], [23], [24], [25]. There are several problems with this approach. First, since there is not a mechanism that binds visual features to form descriptions of object and scene categories, these models often assign the incorrect category label because the feature space fails to provide sufficient discrimination power. Second, extensive training is needed to find an optimal decision boundary in a high-dimensional feature space. Finally, over-fitting can easily occur since the number of training samples is very small relative to the very large number of dimensions of the feature space.

In this paper, we took a different approach to this important visual task. Natural visual scenes consist of objects of various physical properties that are arranged in 3D space in a variety of ways. When projected onto the retina, visual scenes entail highly structured statistics, occurring over the full range of natural variations in the world [26], [27], [28], [29]. To deal efficiently with this full range of natural variations, the visual system may generate percepts according to the PDs of visual variables underlying any stimulus. Thus, we proposed that the visual system performs statistical inference based on a set of coarse hierarchical probabilistic models of natural object and scene categories to achieve rapid scene categorization. To test this hypothesis, we focused on a special case of rapid scene categorization, i.e., categorizing natural scenes with or without animals, and street scenes with or without cars. Current computer vision algorithms are not very successful in performing this task since the context, animals, cars, locations of animals or cars can vary greatly from one scene to another. We developed PDs of object categories that reflect object geometry, spatial configuration of object parts, and natural object structures (i.e., concatenations of a set of local object features). Remarkably, we found that the model localized and categorized animals in natural scenes and cars in street scenes with a near human-level performance.

## Results

### A hierarchical probabilistic model of images of objects in natural scenes

**Figure 1:**
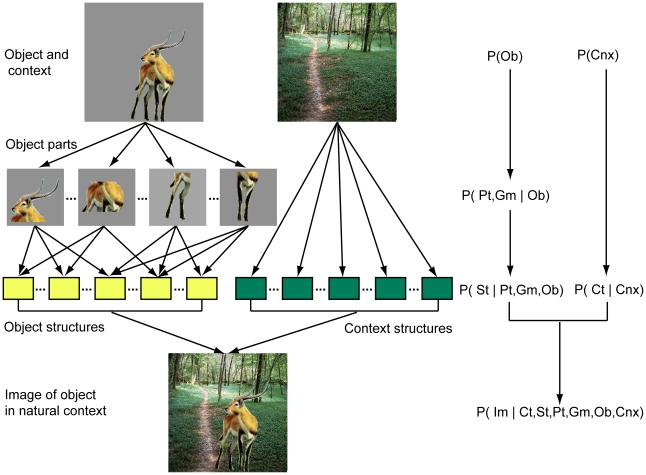
Hierarchical probabilistic model of object categorization in natural scenes. Object category is modeled as a composition of a set of geometrically related parts and each part is represented by a PD of a set of natural object structures. Natural context is modeled by a PD of natural context structures. Object categorization in natural scenes is performed as statistical inference. All the PDs were estimated from natural objects and context.

Our goal in this paper was to develop a model that locates and categorizes objects in natural scenes. This task (e.g., detecting animals in natural scenes) is especially challenging since object appearance in natural scenes can vary enormously and where the objects are located in the scenes is unknown. To achieve this goal, we developed a hierarchical probabilistic model (Figure 1).

In this model, an object is conceived to consist of multiple parts and each part consist of a set of natural object structures, each of which is a concatenation of local features in a small region of the object. Similarly, natural context consists of a set of natural context structures. An image of any object in natural context is seen as being rendered from a set of object and context structures. Each of the processes shown in Figure 1 can be described by a probabilistic model. We used the following notations:

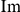
 represents an image,

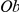
 is an object,

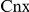
 describes context,

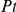
 is a set of object parts,

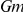
 represents geometric relationship among object parts,

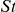
 is a set of object structures,

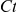
 is a set of context structures.

According to the generative model (Figure 1), we have a probabilistic model of images of objects in natural scenes (Eq. (1)).
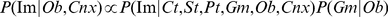
(1)where 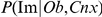 is the probability of an image of a given object in a specific context; 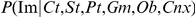 is the probability of an image as being rendered from a set of object and context structures; and 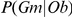 is the probability of the geometry of a given object.

Using Bayes formula, we can then achieve detecting and categorizing objects in natural context via Eq. (2)
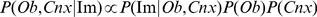
(2)where 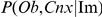 is the posterior probability of an object in an observed image and 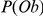 and 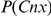 are prior PDs of objects and context.

This model differs from other models of scene categorization [11], [14], [15], [20], [21], [22], [23], [24] in three major ways. First, our model uses explicit structural descriptions of natural objects. In particular, object structures in our model are concatenated to encode objects. Other models usually use a large set of features and do not specify how features are combined to form objects. Second, our model uses hierarchical PDs of natural objects and statistical inference for scene categorization. Other models [20], [21], [22], [23], [24] use extensive training to obtain a decision boundary in a high-dimensional feature space for categorization. Finally, our model localizes and categorizes objects in natural contexts. Other models [21], [22] don't localize objects in natural contexts and thus often erroneously categorize objects by categorizing the context that co-occurs with objects.

To apply this model to the two tasks of rapid scene categorization, i.e., categorizing natural scenes having animals and street scenes having cars, we performed the following five computational steps:

1. Obtaining a set of training samples by manually segmenting animals and cars from the scene datasets;
2. Developing PDs of object geometry in natural scenes;
3. Compiling a set of object structures and developing a PD for each structure;
4. Selecting a set of object structures and developing a joint PD of the selected object structures for categorization;
5. Performing statistical inference to localize and categorize objects in natural scenes.

In the following sections, we describe the results obtained by these steps.

### Coarse PDs of object geometry in natural scenes

**Figure 2:**
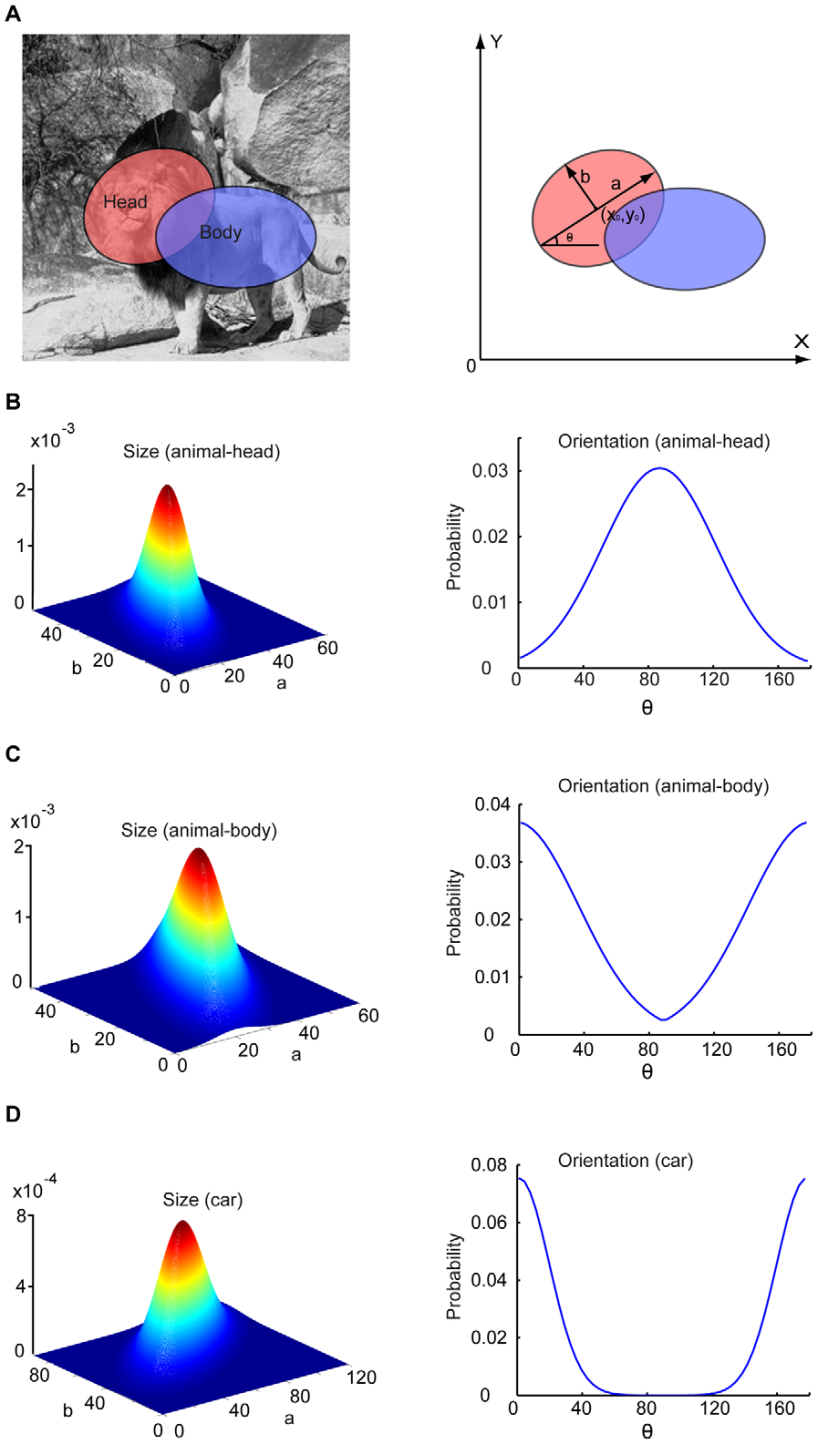
Coarse models of geometry of animals (medium-body animals) and cars in natural scenes. (A), Any animal in natural scenes was modeled by two ellipses, one for the head and one for the body. Any car in natural scenes was modeled by one ellipse. (B), Size (left) and orientation (right) distributions of animal heads in natural scenes. (C), Size (left) and orientation (right) distributions of animal bodies in natural scenes. (D), Size (left) and orientation (right) distributions of cars in natural scenes.

To model human performance on scene categorization, we developed coarse models of object geometry in natural context. We modeled any animal in natural scenes by two ellipses, one for the head and one for the body (Figure 2A). For this purpose, we segmented animals from a set of training scenes by hand. Although current computer vision algorithms can do a decent job on this task (e.g., [30]), we chose to do it manually simply because we need accurate segmentation for compiling object structures (see the following sections). After segmentation, we fitted the histogram of the parameters of the two ellipses to a multi-dimensional Gaussian PD. The distribution of the sizes of animal heads in the dataset of animal scenes had a peak at (35 pixels, 24 pixels) (left panel in Figure 2B). The distribution of the orientations of animal heads had a peak at 87° and a standard deviation of 35° (right panel in Figure 2B). The distribution of the sizes of animal bodies had a peak at (37 pixels, 31 pixels) (left panel in Figure 2C). The distribution of the orientations of animal bodies had a peak at 178° and a standard deviation of 38° (right panel in Figure 2C).

Similarly, we modeled any car in street scenes by an ellipse and fitted the histogram of the parameters of the ellipses obtained from cars segmented manually from a set of street scenes to a multi-dimensional Gaussian PD. The distribution of the sizes had a peak at (78 pixels, 50 pixels) (left panel in Figure 2D). The distribution of the orientations of cars in the dataset of street scenes had a peak at 1° and a standard deviation of 18° (right panel in Figure 2D).

Thus, both animals in natural scenes and cars in street scenes have characteristic statistics in their geometry. We will incorporate these statistics for rapid categorization of natural scenes having animals and street scenes with cars.

### Statistics of natural object structures

We proposed that natural object structures (i.e., concatenations of local features in small regions of images of natural objects) are the units for encoding natural objects and categories for rapid scene categorization. In this proposal, each structure is characterized by a small number of dominant independent components (ICs), obtained by independent component analysis (ICA) [31], [32], and natural variations of each structure by a PD. There are several advantages for using natural object structures: 1) they are more robust than simple features, 2) they take less time to compute than symbolic representations, and 3) they presumably have more descriptive power than simple features including the widely used SIFT features [33]. As it will become clear later on, natural object structures represent spatial concatenations of local features and their PDs (i.e., joint PDs of a set of local features) are more powerful than simple features.

**Figure 3:**
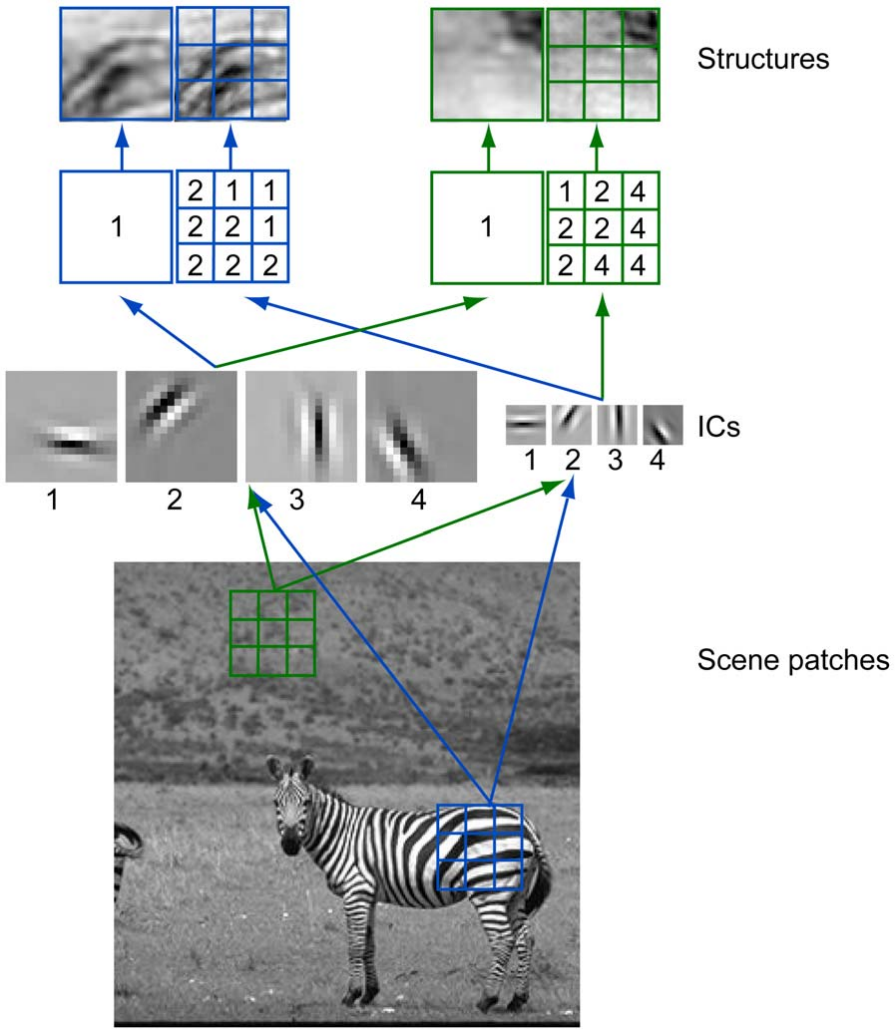
Extracting natural object and scene structures. Each structure is a structured patch compiled from images of natural objects and scenes. To obtain a set of natural object and context structures, we performed ICA on patches of natural scenes and classified the ICs into four orientations. We then sampled a large number of patches from natural scenes and classified each patch as being oriented in one of the four orientations according to the root total square amplitude of the ICs at that orientation. We applied this procedure to a collection of 3×3 small patches and its corresponding 1×1 big patch. A structure was thus a pair of 3×3 structured patches and its corresponding 1×1 structured patch. The structures shown here were the average of all patches that shared same dominant orientational structure at two spatial scales.

For rapid scene categorization, we treated each structure as a structural description of patches of object images at two spatial scales. The coarse structural description was for image patches of 48×48 pixels and the fine structural description was for the 3×3 blocks of the same patches (each block had 16×16 pixels) (see Figure 3 and Materials and Methods). The advantages of using structural descriptions at multiple spatial scales are: 1) they capture structural information of objects at multiple spatial scales in a compact way, and 2) they naturally incorporates scaling invariance, at least to some extent. To derive these structural descriptions, we performed the following four computational steps:

1. Obtaining the ICs of image patches of 48×48 pixels and the ICs of image patches of 16×16 pixels by performing ICA;
2. Clustering the ICs into four clusters, each of which had one of four orientations (0°,45°,90°, and 135°);
3. Computing the root total square amplitudes of the ICs in each of the four clusters for each image patch and block;
4. Describing the structure of each patch and block by the dominant root total square amplitude of the ICs and assigning all image patches that shared the same structural description to the same structure.

Figure 3 shows two structures, one for the zebra and one for the background. The two structures captured the main patterns in luminance variation in the scene patches but did not match them pixel by pixel since the structures shown here were averages of many image patches.

**Figure 4:**
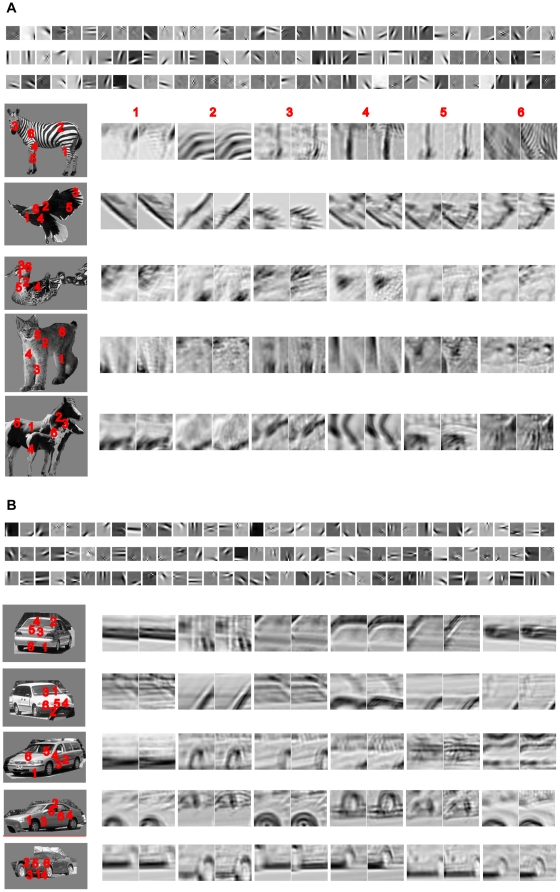
Examples of frequent object structures. The upper panels in (A) and (B) are examples of the ICs of images of animals and cars at a finer scale respectively. Each frequent structure for the 5 animals and cars was the average of patches that shared the same dominant orientation structure at two spatial scales. The numbers indicate the locations of the structures in the animals and cars. The coarse structural description was for image patches of 48×48 pixels and the fine structural description was for the 3×3 blocks of the same patches (each block had 16×16 pixels). Most of the structures at the two spatial scales are similar except some fine details, e.g., the No. 4 and 6 structures of the zebra. The structures at the second scale (3×3 blocks) contain more details than the first scale (48×48 pixels).

As shown in Figure 4, structures compiled from natural objects (i.e., animals and cars) are concatenations of features in small patches on objects. These descriptions are not the same as image patches cropped from objects and range from very simple concatenations (e.g., one or two oriented bars) to very complex concatenations (e.g., texture patterns on animals or cars). The upper panels in Figure 4A and B show examples of the ICs of image patches (16×16 pixels) of animals and cars respectively. The lower panels in Figure 4A show six frequent structures compiled from each of the five animals. Each frequent structure was the average of all patches that shared the same structural descriptions at two spatial scales. The numbers indicate the locations of the structures in the animals. These structures represent coarse but informative descriptions of various parts of the animals, including heads, bodies, legs, necks, wings, and furs. The lower panels in Figure 4B shows six frequent structures compiled from each of the five cars. These structures represent coarse descriptions of various parts of the cars, including screens, windows, tires, roofs, and hoods. Note that since they were averages of many samples, the animal and car structures at the two scales shown here were very similar to each other. These examples, however, are only for illustration purpose and were not used to encode objects. The variations in the features in the scene patches (i.e., ICs) at the two scales can be described by a set of PDs, which contain more information than the average structures shown here. These PDs will be used for object encoding and categorization.

**Figure 5:**
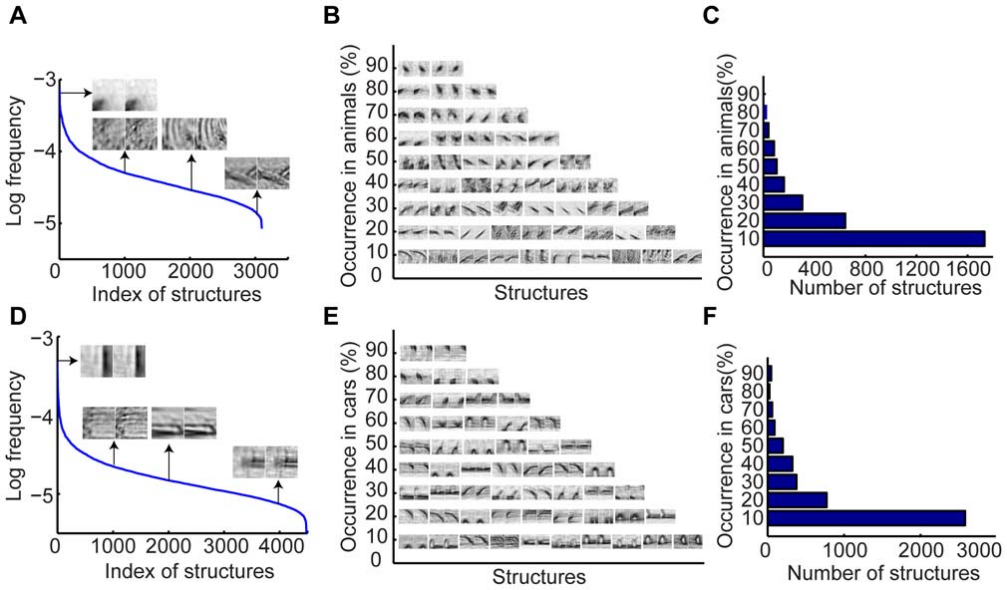
Statistics of object structures. (A), Relative occurring frequency of animal structures. (B), Examples of animal structures. The vertical axis indicates the percentage of the animals in the dataset by which the structures were shared. (C), The total numbers of structures that were shared by different percentage of the animals in the dataset. (D)–(F), Same format as (A)–(C) respectively for car structures.

We then examined the statistics of object structures compiled from a set of animals and cars. We found that simpler structures occur more frequently and are shared by more objects and most structures are shared by only a few objects. Figure 5A shows the normalized frequency of 3,100 structures shared by at least 10% of the animals in the training set. The most frequent structure in animals is a patch with a dark spot at the lower left. The 1,000^th^, 2,000^th^, and 3,000^th^ structures are a fur patch, a patch of zebra strip, and a patch of deer head respectively. Figure 5B and C show examples of structures and the total number of structures shared by different percentage of the animals in the training set respectively. There are only 3 structures shared by 90% of the animals while there are 1,734 structures shared by 10% of the animals.

Figure 5D shows the normalized frequency of 4,481 structures shared by at least 10% of the cars in the training set. The most frequent structure in cars is a vertical bar. The 1,000^th^, 2,000^th^, and 4,000^th^ structures are structured patches of front window, trunk, and front left bump respectively. Figure 5E and F show examples of structures and the total number of structures shared by different percentage of the cars in the training set respectively. There are 48 structures shared by 90% of the cars while there are 2,574 structures shared by 10% of the cars.

In the next section, we will examine the information content of these structures and how to select and combine a set of structures for rapid scene categorization.

### PDs of object structures

**Figure 6:**
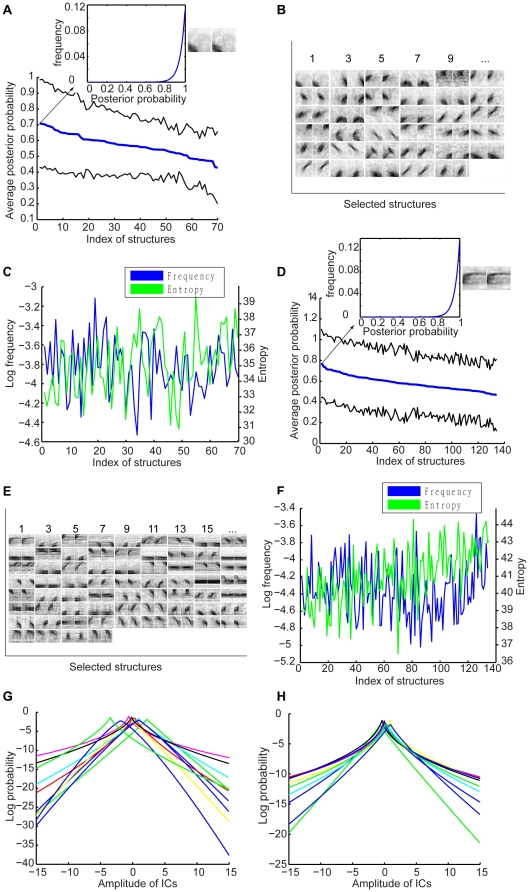
PDs of selected object structures. (A), Average posterior probability of being an animal scene based on each selected structure. The thin lines indicate the standard deviation. The insert shows the PD of the posterior probability of being an animal scene based on the structure. (B), The structures with odd indices shown in (A). (C), Relative occurring frequency and entropy of the 70 selected structures. (D)–(F), Same format as (A)–(C) respectively for car structures. (G), Ten examples of fitted generalized Gaussian PDs of the amplitudes of the ICs of joint probability based on the selected animal structures. (H), Examples (10) of fitted generalized Gaussian PDs of the amplitudes of the ICs of joint probability based on the selected car structures.

We characterized each structure by a 10-dimensional Gaussian PD of the root total square amplitudes of the ICs in the four clusters (one dimension for image patches of 48×48 pixels and one dimension for each of the 3×3 blocks of the image patches). These structures convey a variety of amounts of information about object categories. We selected a set of object structures that were shared by more than 70% of the animals or cars in the training set and performed categorization on segmented animals or cars using each of these structures. Figure 6A shows the performance on categorizing animals. The thick line is the average posterior probability and the thin lines indicate the standard deviation. The insert shows the frequency of the posterior probability based on a structure. The structures indexed by odd numbers in Figure 6A are shown in Figure 6B. Figure 6C shows the relative occurring frequency and differential entropy of the PD of each of these structures. The indices of object structures are the same in Figure 6A and C. There was negative correlation (−0.39) between average posterior probability and differential entropy. These results indicate that object structures shared by more than 70% of the animals in the training set gave rise to relatively good categorization performance.

Figure 6D shows the performance on categorizing cars using individual structures that were shared by more than 70% cars in the training set. The structures indexed by odd numbers in Figure 6D are shown in Figure 6E. Figure 6F shows the relative occurring frequency and differential entropy of the PD of each of these structures. The indices of object structures are the same in Figure 6D and F. As with the animal scenes, there was negative correlation (−0.61) between average posterior probability and differential entropy and object structures that were shared by more than 70% of the cars in the training set gave rise to relatively good categorization performance.

Based on these results, we selected 70 structures for categorizing animal scenes and 134 structures for categorizing street scenes in a random training-testing run (see Materials and Methods). Thus, each selected structure indicated that any given scene had animals or cars at a certain probability (i.e., the posterior probability) and each scene corresponded to a vector of probability conveyed by the set of selected structures. We then need to model the joint distribution of these probabilities. Let 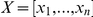 denote these probabilities based on the selected structures (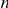 was 70 for animal categorization and 134 for car categorization). We performed ICA on the data of 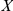 for a set of training images to obtain a set of ICs and fitted the histogram of the amplitude of each of these ICs to a generalized Gaussian PD (∼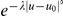, where 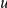 is the amplitude of the IC, 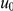 is the mean of the amplitude, 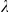 is a positive constant, and 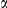 is an exponent). We then modeled the joint PD of the selected structures as a product of generalized Gaussian PDs obtained in this way. Figure 6G and H show examples of the PDs of the amplitudes of the ICs of 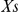. These generalized Gaussian PDs had exponents ranging from 0.50 to 1.28 and 0.43 to 1.45 for animal and cars respectively.

We will use these joint PDs of the selected structures to perform scene categorization. It will become clear in the following sections that near human-level categorization performance can be achieved by combining a small number of selected object structures, each of which only gave rise to low categorization performance.

### Categorizing natural scenes with animals

Detecting animals in natural scenes is a challenging task for which no successful computer vision algorithms have been developed. To apply our model to this task, we need to localize and categorize animals in natural scenes in an integrated way. We achieved this goal in three steps.

**Figure 7:**
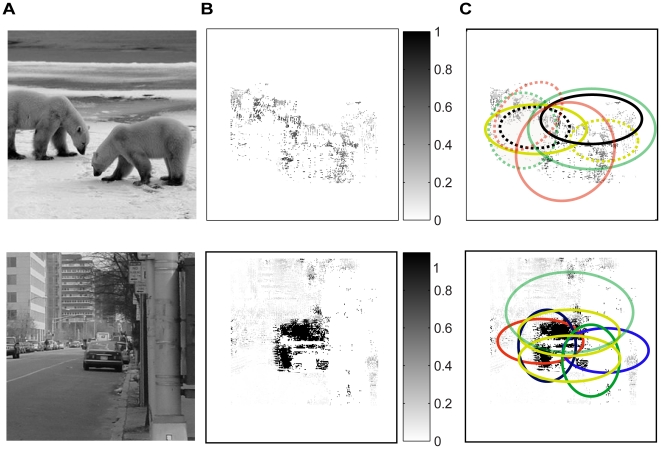
Object localization in natural scenes. (A). Two input scenes. (B). Probability maps, i.e., the probability of a scene patch of 48×48 pixels being an animal or car at each pixel. (C). Object candidates (i.e., the ellipses) sampled from the PDs of coarse object geometry estimated from training scenes were overlaid on the probability maps so that the object candidates covered most pixels that had high probability. The dashed and sold ellipses in upper panel were for animal heads and bodies respectively.

First, we calculated the posterior probability of being an animal of a patch of 48×48 pixels centered at each pixel in a testing scene using the PDs of structures developed earlier to obtain a map of posterior probability. Figure 7A shows two testing scenes and Figure 7B shows the corresponding maps of posterior probability.

Second, we sampled 300 object candidates (i.e., 300 sets of ellipses) from the coarse geometrical PDs of animals and projected the ellipses to the testing scene to cover most pixels at which the posterior probability was greater than 0.6. The upper panel in Figure 7C shows several pairs of ellipses for an animal scene (dashed ellipses were for the animal head and solid ellipses were for the animal body). The lower panel in Figure 7C shows several ellipses for a street scene.

Third, we computed the posterior probabilities of being an animal of the 300 object candidates and selected the candidate that had the highest posterior probability determined by the joint PD of the 70 selected animal structures. We then categorized the testing scene as a scene with animals if the posterior probability was greater than 0.5.

**Figure 8:**
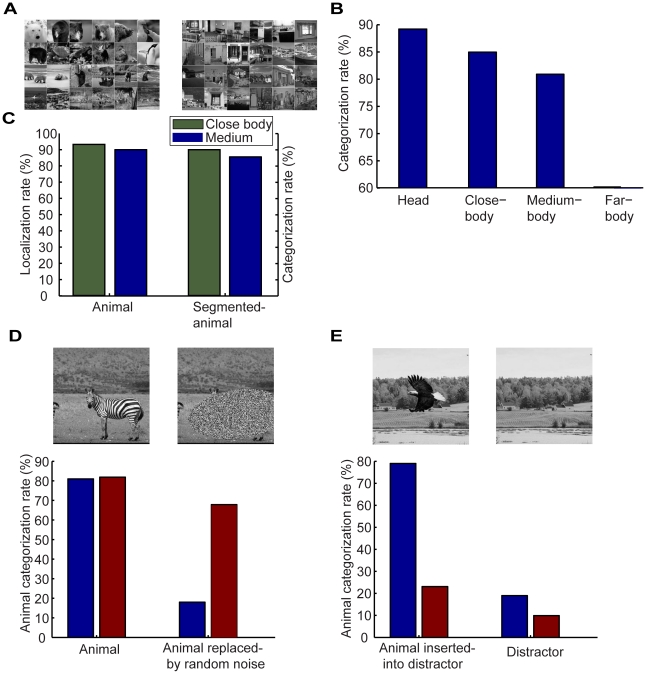
Categorizing animals in natural scenes. (A), Examples of four sets of animal scenes, i.e., head, close-body, medium-body, and far-body, and examples of distractors. (B), Performance of categorizing natural scenes with animals. (C), Performance of localizing animals in natural scenes and categorizing animals segmented from natural scenes for close-body and medium-body. (D), Performance of categorizing scenes having animals and with animals being replaced by random noise. (E), Animal categorization in scenes where animals were inserted into distractors. In (D) and (E), red bars show the results of the Serre et al model on medium-body scenes.

Figure 8A shows examples of animal scenes and distractors in the dataset. These examples make the challenge of detecting animals clear: there are a variety of animals and large variations in the pose, size, texture, and position of the animals in the scenes. Figure 8B shows the performance of our model on this task for four sets of animal scenes, each of which corresponds to a certain viewing distance from the camera, i.e., head, close-body, medium-body, and far-body. The categorization rate is 89%, 85%, 81%, and 60% for the head, close-body, medium-body, and far-body scenes respectively. Thus, the performance degraded for the objects at far distances from the camera. This is because animals occupied fewer image pixels in the medium-body and far-body scenes.

These results are consistent with human performance. In a study done by Serre et al [21], human subjects were asked to detect scenes in which there were animals and identified 92%, 88%, 82%, and 63% of the animal scenes for the head, close-body, medium-body, and far-body conditions respectively. We also tested the model using the animal structures at either the coarse or fine spatial scale. The performance was bad if only the animal structures at the coarse scale were used. The result given by the structures at the fine scale was about 5 percent worse than that by the joint animal structures at the two spatial scales. In the following, we will focus on the close-body and medium-body animal scenes since there is little context in the head animal scenes and too much context in the far-body scenes.

Our model also located the animals in these scenes. If the model identified more than 55% of the pixels occupied by animals in any scene, we called it a case of correct localization. Figure 8C shows the results. The localization rate was 92% and 89% for the close-body and medium-body scenes respectively. As a control, we also examined the performance of our model on categorizing animals segmented from the scenes. For this purpose, we selected an equal number of contextual scene patches and segmented animal images. As shown in Figure 8C, the categorization rate was 90% and 85% for the close-body and medium-body animal scenes respectively. Thus, the categorization rate became lower because localization was not perfect in this case. Note that we did not attempt to precisely localize animals in the scenes but rather to localize them at a reasonable precision to achieve rapid scene categorization.

We examined how rapid our model accomplished this task of scene categorization. Extracting features (i.e., compiling animal structures) from a 256×256 grayscale scene took 73 s in our model and 74 s in the Serre et al model [21], a representative of many models of scene categorization. Categorizing a 256×256 grayscale scene took 192 ms in our model and 157 ms in the Serre et al model. We obtained these results using the same hardware (Intel E8500 3.16 GHz processor with 8G memory) and software (Matlab, Version 7.8.0.347). Thus, in terms of computational time, our model is comparable to the Serre et al model, and rapid scene categorization can be achieved.

One important feature of our model is that, in contrast to many other models for scene categorization where categorization is not performed based on explicit models of object categories in natural scenes, categorization is based on the PD of animals along a set of structures compiled from animals. Our model would thus necessarily categorize scenes with animals regardless of any other features in the scenes. To test this prediction, we did two manipulations, replacing animals with random noise in scenes with animals and inserting animals into distractors (scenes without animals). We used all the medium-body animal and distractor (non-animal) images in the dataset for these two manipulations, i.e., 150 animal and 150 distractor (non-animal) scenes. As expected, when animals in the scenes were replaced by random noise, our model did not categorize the altered images as animal scenes (the blue bars in Figure 8D). Similarly, when animals were inserted into the distractor scenes, our model categorized the altered images as scenes with animals (not as distrastors without animals; the blue bars in Figure 8E).

This prediction does not hold for other models in which categorization is predicted by a decision boundary in a high-dimensional feature space but not PDs of object categories. For example, when the animals in the scenes were replaced with random noise, the Serre et al model [21] categorized them as scenes having animals (the red bars in Figure 8D). Conversely, when animals were inserted into the distractor images, the Serre et al model categorized them as distractors without animals (the red bars in Figure 8E). These results suggest that the Serre at al model essentially uses contextual features that co-occur with animals to categorize scenes when animals do not occupy large portions of the scenes.

Why, then, if the context were the same in the animal scenes and distractors in the database used to test the model, as one would certainly assume, would the Serre et al model categorize distractor scenes with animals inserted as scenes not having animals? We have found several reasons for this peculiar behavior. First, the animal and distractor scenes in the dataset were actually very different. Half of the distractors were artificial scenes (e.g., cities, streets, and houses) and the other half were natural scenes that appeared different from the animal scenes. Second, there is no control on the statistics of the contextual features in the model. During training, the model can pick up any feature combination among the ∼6,000 features used (some of the selected features are from animals but most of them are from the context when animals do not occupy large portions of the scenes) to perform the animal vs. no-animal classification. Finally, since the model needs extensive training to set the weights for the features and to obtain a decision boundary in the high-dimensional feature space, once trained on scenes with certain contextual features, it cannot generalize to scenes with different contextual features. Thus, based largely on the different contextual information and due to the poor generalization ability, the Serre et al model, after trained, categorized animal scenes as scenes having animals, distractor scenes as scenes not having animals, animal scenes with animals replaced by random noise as scenes having animals, and distractor scenes with animals inserted as scenes not having animals.

The results shown in Figure 8 are important for several reasons. First, only 70 animal structures were used in our model, a very small fraction of the ∼6,000 image features used in the Serre et al model. The good performance provided by our model suggests that a small number of structures are sufficient for near human-level scene categorization as long as each structure represents a compact concatenation of local features. Second, we used a subset of the scenes to estimate the PDs but did not obtain a decision boundary in a high-dimensional feature space during training. In the Serre et al model, extensive training (i.e., updating a large number of parameters) was needed to obtain an animal vs. no-animal boundary in a high-dimensional feature space. Finally, our model identified the locations of the animals in natural scenes and categorized them, while the Serre et al model did not make a distinction between animal features and contextual features.

### Categorizing street scenes with cars

**Figure 9:**
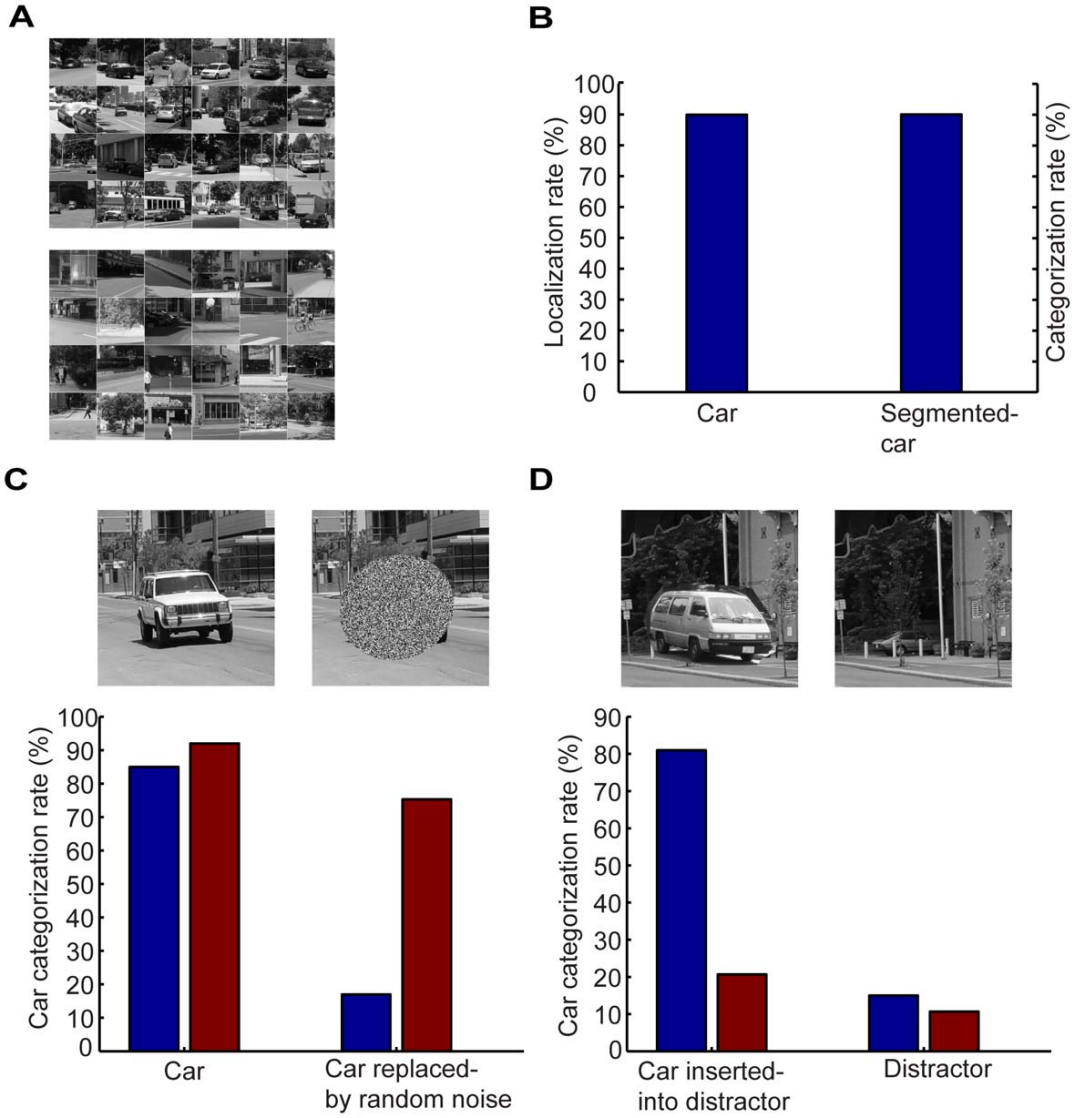
Categorizing cars in street scenes. (A), Examples of street scenes having cars and examples of distractors. (B), Performance of localizing cars in street scenes and categorizing cars segmented from street scenes. (C), Performance of categorizing street scenes having cars and with cars being replaced by random noise. (D), Car categorization in scenes where car were inserted into distractors. In (C) and (D), red bars show the results of the Serre et al model.

We also used our model to detect cars in street scenes. As with animal scenes, detecting cars in street scenes is challenging since there are a variety of cars and trucks, large variations in pose, size, and position of cars in street scenes, and a variety of background clutter (Figure 9A). For this task, we selected 134 frequent car structures. The blue bars in Figure 9B–D show the performance of our model. Our model categorized 85% of the scenes having cars correctly. We also did two manipulations, replacing cars with random noise in scenes having cars and inserting cars into distractors (scenes without cars). We use all the car and non-car images in the database for these two manipulations, i.e., 600 car and 600 distractor (non-car) images. As with the case of animal categorization, our model reported that there were no cars in the first case and that there were cars in the second case (the blue bars in Figure 9C and D). In contrast, the Serre et al model categorized them as scenes having cars in the first case and as scenes not having cars in the second case (the red bars in Figure 9C and D). Therefore, the Serre et al model does not categorize cars per se, but essentially uses contextual features that co-occur with cars to categorize street scenes when cars do not occupy large portions of the scenes.

Our model also found where the cars were located in the scenes. If the model identified more than 55% of the pixels occupied by cars in any scene, we called it a case of correct localization. The localization rate was 90% (Figure 9B). As a control, we also examined the performance of our model on categorizing cars segmented from the scenes. For this purposes, we selected an equal number of contextual scene patches and images of segmented cars. As shown in Figure 9B, the categorization rate was 90%, a result that was better than categorizing cars in street scenes. Thus, in this case, the categorization rate became lower because localization was not perfect.

Thus, with a small number of structures compiled from cars and a coarse hierarchical PD of cars, localizing and categorizing cars in street scenes can be achieved at a high rate of success. The model requires minimal training since the needed PDs are very simple and thus avoids over-fitting and categorizing cars by categorizing context, as in other models.

## Discussion

### PDs of object and scene categories for rapid scene categorization

Our results suggest that, at least for some categorization tasks, explicit (albeit coarse), PDs of categories of objects and scenes are useful and rapid scene categorization can be performed via Bayesian inference based on these PDs. This is a conceptual break from several popular ideas. One idea is that the visual system may use a “short cut”, i.e., a set of statistics of some global image features (e.g., energy of spectral at low spatial frequency [11], [14] and histograms of some low-level features [20]) to achieve rapid scene categorization. Another idea is to use a set of hierarchically organized artificial neurons to encode a large set of image features and to achieve scene categorization by drawing a decision boundary in this high dimensional feature space through extensive training. One such model [21], [22] has been based on many years' research on computational modeling of object recognition. As demonstrated in Figures 8 and 9, for tasks similar to categorizing scenes having animals or scenes having cars, these and other similar models actually categorize the contextual features that co-occur with animals or cars in natural scenes when animals or cars do not occupy large portions of the scenes.

Our model is different from other models in that: 1) coarse hierarchical PDs of natural object categories are used that include PDs of object geometry and spatial configuration of object parts; 2) natural object categories are encoded by a set of object structures, each of which conveys an amount of information about the encoded object category; 3) object localization and categorization is performed in an integrated way. Since, for rapid scene categorization, precise segmentation and detailed object descriptions are not needed, the computation in our model (i.e., compiling object structures, estimating the needed PDs, and performing Bayesian inference) is simple and fast. As shown in Figures 8 and 9, near human-level performance can be readily achieved by our model.

### Natural object structures as units for object encoding

Object perception (e.g., recognition and categorization) has been the focus of neuro-physiological studies and computational modeling in the last 30 years [34], [35]. Neuro-physiological studies have revealed much information about the processing of object encoding from V1 to V2, V4, and to the IT cortex [36], [37], [38], but computational modeling has not yet incorporated this information [25], [39]. Despite these efforts, we know little about the basic units and computations for object encoding and recognition.

We demonstrated that a small number of natural object structures are sufficient for encoding complex object categories such as animal and car. In this encoding scheme, each object structure is a concatenation of local image features and conveys an amount of information about the object and category; general structures are shared by more objects and specific structures are shared by only a few objects in the category. Thus, a selected combination of general and specific structures could encode information for both object and category and a PD on these object structures could quantitatively characterize natural variations of both objects and categories. It is conceivable that visual neurons encode natural object structures and their PDs and perform statistical operations on these PDs to achieve object perception. Indeed, current observations on the neural processing of object perception (e.g., neuronal tuning for complex features, views, object categories, scale, position, and pose tolerance, and feature columns in the IT area) [36], [37], [38] can be interpreted as encoding of object structures in a hierarchical way by neurons in the ventral pathway. In this interpretation, preferred stimuli are object structures and neuronal responses indicate the probabilities of object structures. Populations of neurons can thus encode a large of number of object structures and virtually infinite number of objects.

We should point out that, although the work presented here suggests a novel concept of visual information processing (i.e., even for rapid scene categorization, coarse, hierarchical probabilistic encoding of natural object categories are needed), the work is only a computational model. How to map the natural object structures and their PDs and the statistical operations in the model to neuronal response properties, neural circuitry, and neural dynamics is essentially unknown. Indeed, in its current form, our model has no temporal component and no direct physiological correlates and thus the time required to implement such a model in vivo is unknown.

### Future directions

The model developed here can be extended and refined in several ways. First, the model can be extended to categorize multiple categories of natural scenes. There is now a large dataset of more than 900 categories of natural scenes, including coasts, rivers, lakes, forest, plains, mountains, landscapes, countries, and deserts. Tested on this dataset, the performance of the best current model for natural scene categorization is ∼40% [40]. To extend our model, a large set of natural object and scene structures are needed and a hierarchical PD is needed for each scene category. It remains to be seen how far our model can go in comparison to human performance. Second, parameterized low-dimensional PDs can be developed to model natural object and scene structures more precisely. Finally, fast algorithms can be developed to achieve better object localization in natural scenes.

## Materials and Methods

### Databases

The dataset of animal scenes and implementation of Serre et al model were downloaded from the Center for Biological & Computational Learning at MIT (http://cbcl.mit.edu/software-datasets/index.html). The dataset contains 600 gray-scale images of a variety of animals (including mammals, birds, fish, insects, and reptiles) in natural scenes and a set of distractor scenes including 300 natural scenes and 300 artificial scenes (Figure 8A). The sizes of the images are 256×256. This dataset have four subsets of scenes, corresponding to a certain viewing distance from the camera, i.e., head, close-body, medium-body, and far-body.

The dataset of street scenes include 600 images of sedans, jeeps, trucks, SUVs, and buses and 600 images of street scenes without cars (Figure 9A). The sizes of these images are 256×256. These images were cropped from a set of images of 1280×960 pixels with random offsets.

We used local contrast in these gray-scale images as inputs in our analysis. We calculated Michelson contrast using a circular center-surround configuration. The radius of the center was 2 pixels and the radius of the surrounding circle was 4 pixels. For far- body animal scenes, the radius of the center and surrounding circle was 1 pixel and 2 pixels respectively.

### Training and testing

We split each of the datasets into two halves, one for training and one for testing. In order to compile a set of object structures and estimate their PDs, we rendered 50 images from each scene in the training sets by performing affine transform and adding white noise. We then selected object structures that occurred frequently in these rendered scenes and estimated the PDs of object structures.

For any input scene, we sampled 300 object candidates from the PD of object geometry and parts estimated in the training step and projected them onto a test scene to cover most pixels at which the posterior probability was greater than 0.6. We then estimated the posterior PDs of these 300 candidates and selected the candidate that had the maximal posterior probability. We repeated this procedure 20 times (i.e., 20 random splits of training and testing sets) and obtained the average categorization rate.

To compare our model with others, we did two manipulations, replacing animals with random noise in scenes with animals and inserting animals into distractors (scenes without animals). We used all the medium-body animal and distractor (non-animal) images in the database for these two manipulations, i.e., 150 animal and 150 distractor (non-animal) images for medium-body. Similarly, we used all the car and non-car images in the database for these manipulations, i.e., 600 car and 600 distractor (non-car) images.

### PDs of object geometry in natural scenes

We modeled any animal in natural scenes by two ellipses, one for the head and one for the body (Figure 2A shows the result of medium-body animals). The rationale was to model coarse object geometry for rapid categorization. We segmented animals from the scenes in the training set manually and fitted the animals to two ellipses and obtained the parameters of the ellipses. We then fitted the histogram of the parameters of the ellipses to a multi-dimensional Gaussian PD (Figure 2B and C). For the head and far-body scenes, we only fitted each animal in the scenes to one ellipse.

Similarly, we modeled any car in street scenes by an ellipse and fitted the histogram of the parameters of the ellipses obtained from cars segmented manually from street scenes to a multi-dimensional Gaussian PD (Figure 2D).

### Compiling object structures

Each structure entailed structural descriptions at two spatial scales. At the coarse scale, a structural description was derived for an image patch of 48×48 pixels. At the fine scale, a set of 9 structural descriptions were obtained for the 3×3 blocks of the same image patch (each block had 16×16 pixels) (Figure 3). To compile these structures, we first obtained the independent components (ICs) of image patches of 48×48 pixels and the ICs of image patches of 16×16 pixels sampled from animals or cars that were manually segmented from natural or street scenes in the training sets. We then classified these ICs into clusters according to the orientation of the ICs. To limit the total number of structures, we used only four clusters (i.e., 0°, 45°, 90°, and 135°). Using these clusters, we assigned a structural label at the coarse scale and a structure label at the fine scale to each image patch of 48×48 pixels sampled from animals or cars. A structural label at the coarse scale was the dominant orientation (i.e., the root total square amplitude of the ICs at that orientation was the greatest among the four orientations). A structural label at the fine scale was the dominant orientations in the 3×3 blocks of the same image patch. Finally, we collected all different structures, i.e., structures that had different structural labels at the two spatial scales.

### PDs of object structures and structure selection

We selected object structures for categorization in two steps. In the first step, we selected structures that were shared by more than 70% of the animals or cars in the training set. In the second step, for each of the selected structures, we developed a 10-dimensional Gaussian PD of the root total square amplitudes of the ICs in the four clusters (one dimension for each of the 10 structural labels). Using these PDs, we performed categorization on segmented animals or cars using each of the structures. We selected 70 animal structures and 134 car structures that gave rise to the best categorization performance. Figure 6B and E shows the selected structures obtained in a training-testing run (see Training and testing above).

### Joint PDs of object structures

Each selected structure indicated that any given scene had animals or cars at certain probability and each scene corresponded to a vector of probability. Let 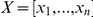 denote these probabilities for each scene (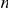 was 70 for animal categorization and 134 for car categorization). Thus, for the scenes in the training sets (see Training and testing above), we obtained a dataset of 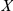. To model the PD of 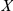, we performed ICA on the dataset of 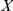 to obtain a set of ICs and fitted the histogram of the amplitude of each of these ICs to a generalized Gaussian PD (∼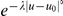, where 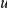 is the amplitude of the IC, 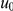 is the mean of the amplitude, 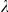 is a positive constant, and 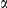 is an exponent. 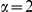 for Gaussian PDs). The PD of 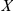 was thus a product of these generalized Gaussian PDs. Figure 6G and H show 10 examples of generalized Gaussian PDs obtained in this way.

### Object localization and categorization

For any test scene, we calculated the posterior probability of a 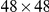 patch centered at each pixel being an animal or a car based on the PDs of the selected structures to obtain a map of posterior probability (Figure 7B). We then sampled 300 objects candidates (i.e., 300 sets of ellipses) from the geometrical PD models of animals or cars and projected the object candidates to the test scene to cover most pixels at which the posterior probability was greater than 0.6. Since we modeled any animal in natural scenes by two ellipses (one for the head and one for the body, Figure 2A), we sampled two ellipses for animals (dashed ellipse for head, solid ellipse for body in Figure 7C).

For each of the 300 object candidates, we first sampled a large number of image patches inside it (the total number of images patches depended on the size of the candidate). We then inserted these patches to the PDs of the selected object structures (70 for animals and 134 for cars) to obtain a vector of posterior probability. Finally, we plugged this vector of probability into the joint PD of object structures to calculate the posterior probability of the candidate being an animal or car. After obtaining the posterior probabilities for the 300 object candidates, we selected the object candidate that had the highest probability of being an animal or a car. If the probability was greater than 0.5, we categorized the selected object candidate as an animal or car; otherwise, we categorized it as a distractor.
